# Left atrial volume index and interleukin-6 as predictors for postoperative atrial fibrillation

**DOI:** 10.1186/s13019-024-02813-9

**Published:** 2024-06-07

**Authors:** Hong Tao, Xiao Shen, Lei Zou, Cui Zhang, Liang Hong

**Affiliations:** Department of Critical Care Medicine, Nanjing First Hospital, Nanjing Medical University, 68 Changle Road, Nanjing, Jiangsu Province 210006 China

**Keywords:** Postoperative atrial fibrillation, Left atrial volume index, Interleukin-6, Cardiac surgery

## Abstract

**Background:**

Postoperative atrial fibrillation (POAF) is a common complication after cardiac surgery. However, the predictive value of single indictor still remains controversial. This study aimed to assess the predictive value of combining preoperative left atrial volume index (LAVI) and postoperative interleukin-6 (IL-6) for POAF in the patients receiving cardiac surgery.

**Methods:**

Patients who admitted to Nanjing First Hospital during the study period between December 2022 and June 2023, and underwent open-heart surgery without a history of atrial fibrillation (AF) were enrolled. The relationships between predictors and POAF were investigated using logistic regression analysis. We determined the combined predictive value of LAVI and IL-6 for POAF by measuring the changes in the area under the receiver operating characteristic curve (AUC) and calculating the net reclassification improvements (NRIs) and integrated discrimination improvement (IDIs).

**Results:**

102 patients were enrolled in this study, and 50 participants developed POAF (49.0%). Patients who experienced POAF had higher levels of preoperative LAVI and postoperative IL-6 than those who did not. Regression analysis revealed that larger LAVI and higher level of IL-6 were independently associated with increased risk of POAF. The combined addition of LAVI and IL-6 to the predictive model resulted in an evident increase in the AUC. Incorporating both LAVI and IL-6 increased IDIs in all models.

**Conclusion:**

Our results demonstrated that combined LAVI and IL-6 achieved a higher prediction performance for POAF.

## Introduction

Postoperative atrial fibrillation (POAF), defined as a new-onset atrial fibrillation (AF) after surgery, is a common complication that occurs following cardiac surgery. POAF is associated with longer lengths of hospitalization and higher mortality. The incidence of POAF has been increasing each year [1].

The underlying pathogenesis of POAF is not fully understood yet. Briefly, POAF results from substrates and trigger factors. Abnormal electrical activity is not maintained in the normal atrium. Susceptible substrates resulting from changes in the atrium lay the foundation of abnormal electrical activity. Volume or pressure overload contributes to these changes. On the base of vulnerable substrates, trigger factors are also needed to induce AF. Trigger factors for POAF include inflammation, oxidative stress and autonomic dysfunction [2]. In conclusion, POAF occurs when trigger factors act on the base of susceptible substrates.

The prediction of POAF is still a common clinical conundrum. Several researches have been developed to predict POAF [3] and found that both preoperative and postoperative factors play important roles in the development of POAF.

Preoperative echocardiographic parameters particularly left atrial volume index (LAVI) is one of the most commonly used variables to predict POAF. Osranek et al. presented that preoperative LAVI was an independent predictor of POAF [4]. However, its predictive value is still controversial. Recently, Kislitsina et al. found that LAVI did not have a high predictive value for POAF [5]. This may be because the study considered only the presence of susceptible substrates, but neglected the impact of trigger factors.

Postoperative inflammatory biomarkers have been also used to predict POAF. Interleukin-6 (IL-6) is the most studied biomarker. Postoperative IL-6 levels were associated with POAF in some studies [6, 7], but not in others [8, 9]. Hence, the predictive value of IL-6 for POAF remains to be further confirmed.

No single predictor has been found to be able to predict the development of POAF well. There is no study that incorporates both preoperative echocardiographic and postoperative biological markers to predict POAF. The aim of our study is to explore the combined predictive value of LAVI and IL-6 for POAF in the patients receiving cardiac surgery.

## Materials and methods

### Study design and population

This study was a prospective case-control study performed at Nanjing First Hospital, a tertiary teaching hospital affiliated to Nanjing Medical University. Institutional ethics approval from the Ethics Committee of Nanjing First Hospital (KY20220518-KS-01) and patient consents were obtained. Adult patients with no history of AF undergoing elective cardiac surgery including CABG and(or) valve surgery during the study period between December 2022 and June 2023 were recruited. The exclusion criteria were as follows: (1) incomplete information; (2) combined with aortic aneurysm, congenital heart disease and cardiomyopathy; (3) during the acute infection; (4) with immune diseases; (5) with poor image quality.

### Cardiac ultrasonography

Critical care physicians with ultrasound training licenses performed echocardiography using Mindray M9 (Shenzhen, China) within two days before cardiac surgery. The patients were examined in the left lateral decubitus position. According to the recommended guideline [10], left atrial volumes were measured by Simpson’s biplane method. Every measurement was taken three times by the same physician, and the mean value was used for calculation. Left atrial volume was indexed to body surface area.

### Serum IL-6 measurement

Peripheral blood samples were obtained for IL-6 detection at the time that patients were transferred to the Cardiovascular Intensive Care Unit postoperatively. The serum levels of IL-6 were detected by quantum dot fluorescence immunoassay, which was completed by Department of Laboratory, Nanjing First Hospital. The kit was purchased from Nanjing Vazyme Medical Technology Co., Ltd and detected by QD-S2000 vitek immune diagnostic assay system.

### Data collection

The following information was collected: (1) demographic data: age, gender, weight, and height; (2) comorbidities: hypertension, diabetes and hyperlipidemia; (3) lifestyle factor: smoking history; (4) oral medications: β-blocker and statin; (5) New York Heart Association(NYHA) classification of cardiac function at hospital admission; (6) type of surgery: CABG, valve surgery, as well as a combined cardiac surgery(CABG and valve surgery); (7)preoperative echocardiographic parameters: LAVI, left atrial diameter(LAD) and left ventricular ejection fraction(LVEF); (8) surgical duration: cardiopulmonary bypass (CPB) time and cross-clamp time; (9) laboratory findings at the admission of ICU: serum creatinine, IL-6, procalcitonin (PCT), white blood cell count, neutrophil count, lymphocyte count, lactate, PaO_2_/FiO_2_, brain natriuretic peptide (BNP) and high-sensitivity C-reactive protein(HS-CRP); (10) ICU admission Acute Physiology and Chronic Health Evaluation II score(APACHE II score) *and* European System for Cardiac Operative Risk Evaluation (EuroScore); (11) use of vasoactive medications during ICU day and vasoactive inotropic score max; (12) outcome indexes: POAF within seven days after operation, intubation time, ICU and hospital stay.

POAF was diagnosed based on ECG records or a written diagnosis of atrial fibrillation, starting from post-operation until the seventh-day post-operation.

### Statistical analysis

Statistical analyses were performed with R language (R version 4.2.1). Independent sample T test was used for measurement data conforming to normal distribution, and the results were expressed as mean ± standard deviation ($\overline{X}\pm s$), while Wilcoxon rank-sum test or Mann–Whitney *U*-test was used for measurement data not conforming to normal distribution, and the results were expressed as median ± interquartile range (IQR). Categorical variables were expressed as percentages and analyzed by χ2 tests.

Logistic regression was used to investigate the association between indictors and POAF, and the odds ratio (OR) with 95% confidential interval (CI) were calculated. We employed restricted cubic splines (RCS) to illustrate relationships between LAVI and IL-6 and POAF respectively. In addition, LAVI and IL-6 were converted into categorical variables and *p* for trend were calculated.

In a prospective, randomized study, investigators found that CPB was strongly associated with the incidence of POAF [11]. In a multicenter study, Akintoye E et al. determined that advanced age, prolonged operative time were independent risk factors for POAF [12]. Although debated, β-blocker is the first-line medication in the prevention of POAF [1]. According to these previous studies and our study, we built 4 predictive models to assess the combined predictive value of LAVI and IL-6 for POAF. Firstly, we quantified the predictive ability of each model using the area under the receiver operating characteristic curve (AUC) and then used Delong’s method to determine the change in AUCs. Additionally, we calculated the indices of net reclassification improvements (NRIs) and integrated discrimination improvements (IDIs) to evaluate the risk reclassification capability of combined indictors.

## Results

### Patient baseline characteristics

**Fig. 1:**
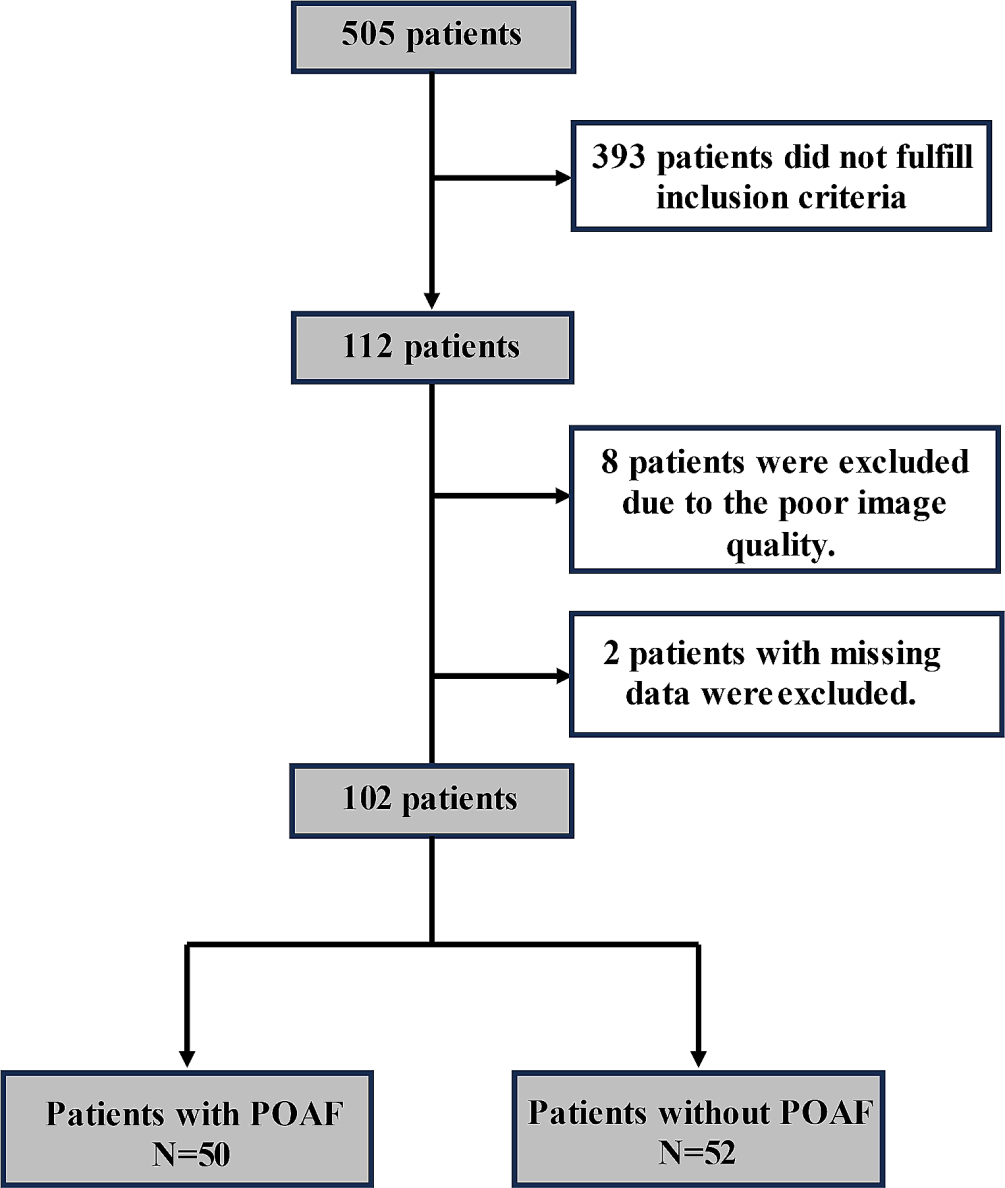
The flow-process diagram. Abbreviations: POAF, postoperative atrial fibrillation

During the study period, 102 patients were finally included for this sutdy. Figure 1 showed the flow-process diagram of patients enrolling. The patient baseline characteristics were summarized in Table 1. POAF occurred in 50 patients finally (49.0%). Patients who suffered POAF had larger preoperative LAD and LAVI than those who did not (LAD: 44 vs. 41 mm, *p* = 0.033 and LAVI: 22 vs. 17 ml/m^2^, *p* = 0.005, respectively). There were significant differences in the NYHA classification of cardiac function during the preoperative period between the two groups (*p* = 0.001). When we evaluated the association between surgical characteristics and POAF, three major surgery characteristics including type of surgery, cardiopulmonary bypass time and cross-clamp time were each significantly associated with the risk of POAF. Postoperative clinical data were displayed in Table 2. Patients with POAF had higher levels of IL-6 and PCT than those without POAF (IL-6: 92.29 vs. 63.40 pg/ml, *p* = 0.004 and PCT: 0.08 vs. 0.05 ng/ml, *p* = 0.013, respectively). There was significant difference in EuroScore on the first day in ICU (*p* = 0.008). When we compared the postoperative cardiac echocardiography five days after operation, two major variables including LAD and LVEF were each significantly different between the two groups. We observed that patients who developed POAF used a greater dose of vasoactive agents than those who did not (*p* = 0.002). Length of ICU stay and hospital stay as well as duration of mechanical ventilation time were longer among those patients with POAF, although without statistically significance.

**Table 1:** Baseline characteristics, preoperative cardiac ultrasonography and surgical information

	Patients with POAF (*N* = 50)	Patients without POAF (*N* = 52)	*P*-value
Age, years	67.00 [59.00, 71.75]	61.00[54.75, 71.00]	0.189
BMI, kg/m^2^	24.22 ± 3.65	24.86 ± 3.01	0.333
Male sex	34 (68.00%)	37 (71.15%)	0.896
Smoking history	24 (48.00%)	23 (44.23%)	0.528
Comorbidity			
Hypertension	25 (50.00%)	30 (57.69%)	0.562
Diabetes mellitus	19 (38.00%)	23 (44.23%)	0.661
Hyperlipidemia	4 (8.00%)	6 (11.54%)	0.789
Oral medications			
β-blocker	27 (54.00%)	25 (48.08%)	0.689
Statin	20 (40.00%)	24 (46.15%)	0.669
Echocardiographic parameters			
Preoperative LAVI, ml/m^2^	21.77 [16.09, 28.70]	16.74 [13.08, 22.01]	0.005
Preoperative LAD, mm	43.70 ± 5.50	41.46 ± 5.00	0.033
Preoperative LVEF, %	61.00 [51.25, 64.75]	62.00[58.50, 65.00]	0.332
NYHA			
Class II	14 (28.00%)	32 (61.54%)	0.001
Class III	36 (72.00%)	20 (38.46%)	
Type of surgery			
CABG	17 (34.00%)	33(63.46%)	0.009
Valve surgery	19 (38.00%)	13(25.00%)	
Valve surgery	19 (38.00%)	13(25.00%)	
Combined surgery	14 (28.00%)	6(11.54%)	
CPB time, min	117.50 [82.25, 157.75]	88.00 [78.75, 138.25]	0.062
Cross-clamp time, min	84.50 [57.25, 119.00]	59.50 [51.50, 75.25]	0.011

*Abbreviations* POAF, postoperative atrial fibrillation; BMI, body mass index; LAVI, left atrial volume index; LAD, left atrial diameter; LVEF, left ventricular ejection fraction; NYHA, New York Heart Association; CABG, coronary artery bypass graft; CPB, cardiopulmonary bypass

**Table 2:** Postoperative data

	Patients with POAF (*N* = 50)	Patients without POAF (*N* = 52)	*P*-value
Laboratory findings on the first day in ICU			
Creatinine, µmol/L Interleukin-6, pg/mL Procalcitonin, ng/mL High-sensitivity C-reactive protein, mg/L Brain natriuretic peptide, pg/mL Lactate, mmol/L PO_2_/FiO_2_ ratio, mmHg White blood cell count, *10^9/L Neutrophil count, *10^9/L Lymphocyte count, *10^9/L Platelet count, *10^9/L	73.45[63.33,86.10] 92.29[62.57,123.39] 0.08 [0.05, 0.20] 1.60 [0.78, 2.88] 105.00[36.00,433.25] 1.35 [0.80, 2.20] 242.10[185.63,328.28] 10.39 [8.52, 12.35] 9.27 [7.64, 11.22] 0.53 [0.38, 0.69] 118.00 [91.75, 139.75]	74.45 [65.70, 87.53] 63.40 [52.49, 91.77] 0.05 [0.04, 1.00] 1.38 [0.570, 2.16] 77.50[17.00,270.50] 1.30[0.80, 1.73] 271.50[220.83,338.55] 10.89 [9.20, 13.85] 9.93 [8.23, 12.19] 0.54 [0.40, 0.80] 123.50 [93.50, 145.00]	0.569 0.004 0.013 0.501 0.201 0.583 0.322 0.300 0.246 0.447 0.595
Vital signs at ICU admission			
Heart rate, beats/min Mean arterial pressure, mmHg Central venous pressure, mmHg	100.00 [91.25, 109.75] 96.00 [91.00, 102.75] 11.00 [9.00, 13.00]	102.00 [92.50, 111.00] 97.50 [94.00, 102.00] 11.00 [9.75, 12.25]	0.753 0.302 0.590
APACHE *II score* on the first day in ICU	12.00 [9.00, 15.00]	12.00 [9.75, 13.25]	0.457
EuroScore on the first day in ICU	6.00[5.00, 7.00]	5.00[4.00, 6.00]	0.008
Echocardiographic parameters			
Postoperative LAD, mm Postoperative LVEF, %	39.64 ± 5.45 60.50 [49.25, 63.00]	37.09 ± 4.33 62.00[59.00, 64.00]	0.010 0.039
Use of vasoactive agents during ICU stay	40 (80.00%)	36 (69.23%)	0.308
Vasoactive-inotropic score max	5.00 [2.00, 13.00]	3.00 [0.00, 5.00]	0.002
Intubation time, hours	9.00 [6.50, 10.58]	8.25[6.79, 10.53]	0.514
Length of ICU stay, hours	29.95 [20.00, 63.58]	21.85 [20.30, 39.84]	0.392
Length of hospital stay, days	20.00 [15.25, 25.75]	18.00 [16.00, 22.00]	0.208

*Abbreviations* POAF, postoperative atrial fibrillation; ICU, intensive care unit; PaO_2_, partial pressure of arterial oxygen; FiO_2_, fraction of inspiratory oxygen; APACHE II score, Acute Physiology and Chronic Health Evaluation II score; EuroScore, European System for Cardiac Operative Risk Evaluation; LAD, left atrial diameter; LVEF, left ventricular ejection fraction

### The association between indictors and POAF

In the multivariate logistic regression analysis (Table 3), larger preoperative LAVI (OR, 1.10, 95%CI, 1.02–1.20, *p* = 0.02), higher levels of postoperative IL-6 (OR, 1.02, 95%CI, 1.01–1.04, *p* = 0.018) and increased NYHA cardiac function (OR, 3.42, 95%CI, 1.27–9.77, *p* = 0.017) were independently associated with an increased risk for POAF. In addition, we employed RCS to illustrate relationships between LAVI and IL-6 and POAF respectively, as shown in Fig. 2A and B. The OR increased evidently when the LAVI reached approximately 29ml/m^2^ while the OR increased significantly when the level of IL-6 reached approximately 59pg/ml. As a result, RCS demonstrated that LAVI and IL-6 were associated with POAF significantly (*p* = 0.014 and *p* = 0.016, respectively). Table 4 showed the results of the trend test. It indicated that in patients with preoperative LAVI ≤ 29ml/m^2^, the risk of developing POAF did not increase with the increase of IL-6 levels (*p* = 0. 11) while in patients with preoperative LAVI > 29ml/m^2^, the risk of developing POAF increased in accord with the rise of IL-6 levels (*p* = 0.024). When we converted IL-6 from numeric variables to 4-category variables based on their quartiles, the same conclusion still held.

**Table 3:** Association between indictors and POAF

	Univariate logistic			Multivariate logistic		
	OR	95%CI	*P*-value	OR	95%CI	*P*-value
LAVI	1.09	1.03,1.15	0.002	1.10	1.02,1.20	0.020
Interleukin-6	1.01	1.00,1.03	0.010	1.02	1.01,1.04	0.018
LAD	1.09	1.01,1.18	0.037	0.96	0.86,1.07	0.445
NYHA						
Class II Class III	Ref. 4.11	Ref. 1.82,9.69	Ref. < 0.001	Ref. 3.42	Ref. 1.27,9.77	Ref. 0.017
Type of surgery						
CABG Valve surgery Combined surgery	Ref. 2.84 4.53	Ref. 1.15,7.25 1.53,14.82	Ref. 0.026 0.008	Ref. 2.08 2.25	Ref. 0.64,6.91 0.38,14.1	Ref. 0.223 0.374
CPB time	1.01	1.00,1.02	0.062	0.99	0.97,1.01	0.281
Cross-clamp time	1.02	1.00,1.03	0.011	1.01	0.99, 1.03	0.345
EuroScore	1.42	1.08,1.94	0.018	1.17	0.85, 1.68	0.349
Procalcitonin	5.53	0.95,110.98	0.200	1.12	0.24, 22.7	0.939

*Abbreviations* POAF, postoperative atrial fibrillation; EuroScore, European System for Cardiac Operative Risk Evaluation; LAD, left atrial diameter; LVEF, left ventricular ejection fraction; LAVI, left atrial volume index; CABG, coronary artery bypass graft; CPB, cardiopulmonary bypass; NYHA, New York Heart Association; OR, odds ratio; CI, confidential interval

**Table 4:** LAVI and IL-6 were converted into categorical variables

		*P* value for trend	IL-6	*P* value for trend
			Q_1_ ≤ 58.37pg/ml	Ref.
LAVI ≤ 29ml/m^2^	IL-6	0.11	Q_2_ 58.37-74.39pg/ml	0.472
			Q_3_ 74.39-109.24pg/ml	0.998
			Q_4_ ≥ 109.24pg/ml	0.997
		*P* value for trend	IL-6	*P* value for trend
			Q_1_ ≤ 58.37pg/ml	Ref.
LAVI > 29ml/m^2^	IL-6	0.024	Q_2_58.37-74.39pg/ml	0.916
			Q_3_ 74.39-109.24pg/ml	0.421
			Q_4_ ≥ 109.24pg/ml	0.025

*Abbreviations* LAVI, left atrial volume index; IL-6, interleukin-6

**Fig. 2A:**
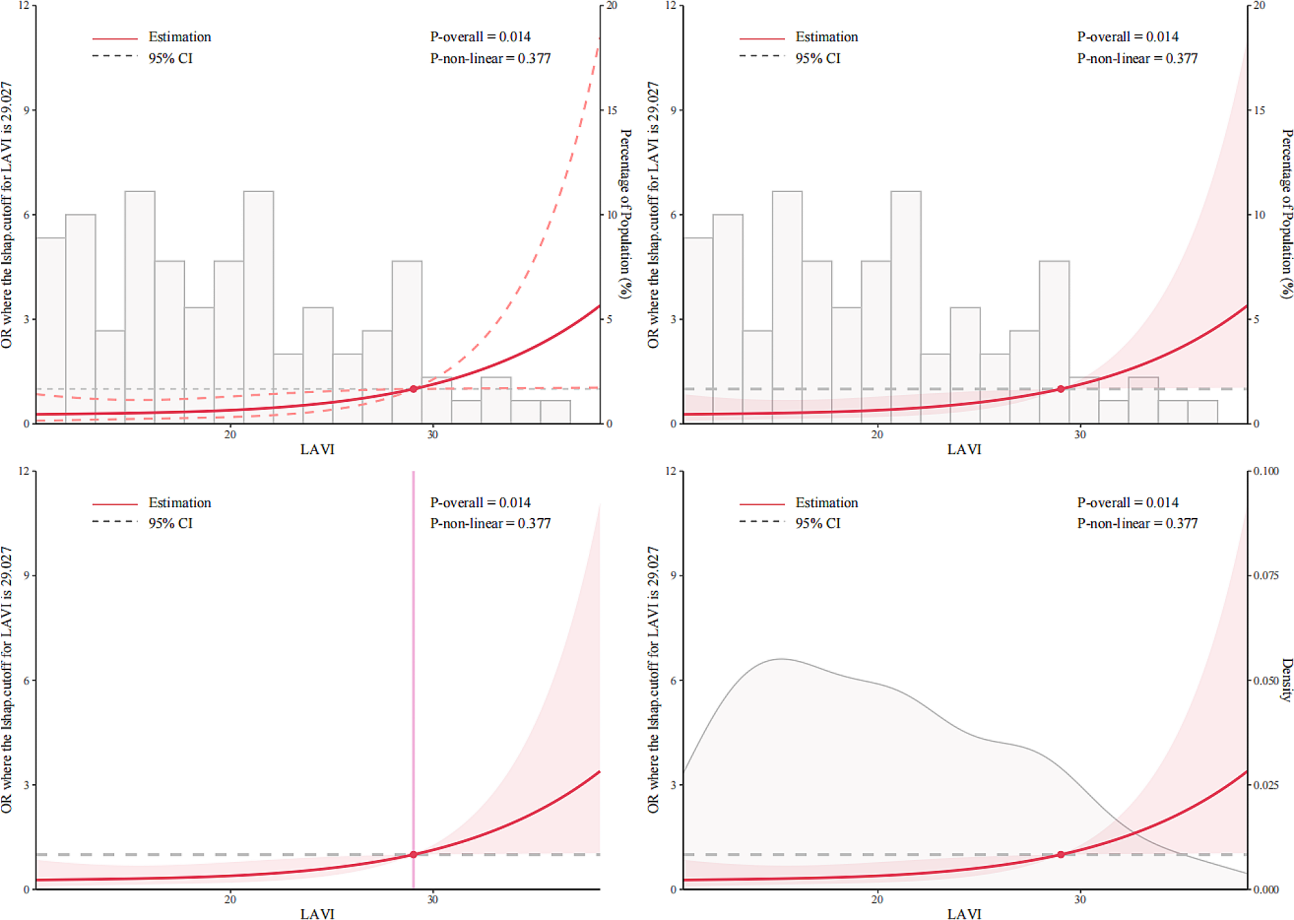
Restricted spline curves for the relationship between LAVI and POAF in cardiac surgery patients. The red bold line denotes the odds ratio, while the shaded area represents the 95% confidence intervals. *Abbreviations* POAF, postoperative atrial fibrillation; LAVI, preoperative left atrial volume index; CI, confidence interval

**Fig. 2B:**
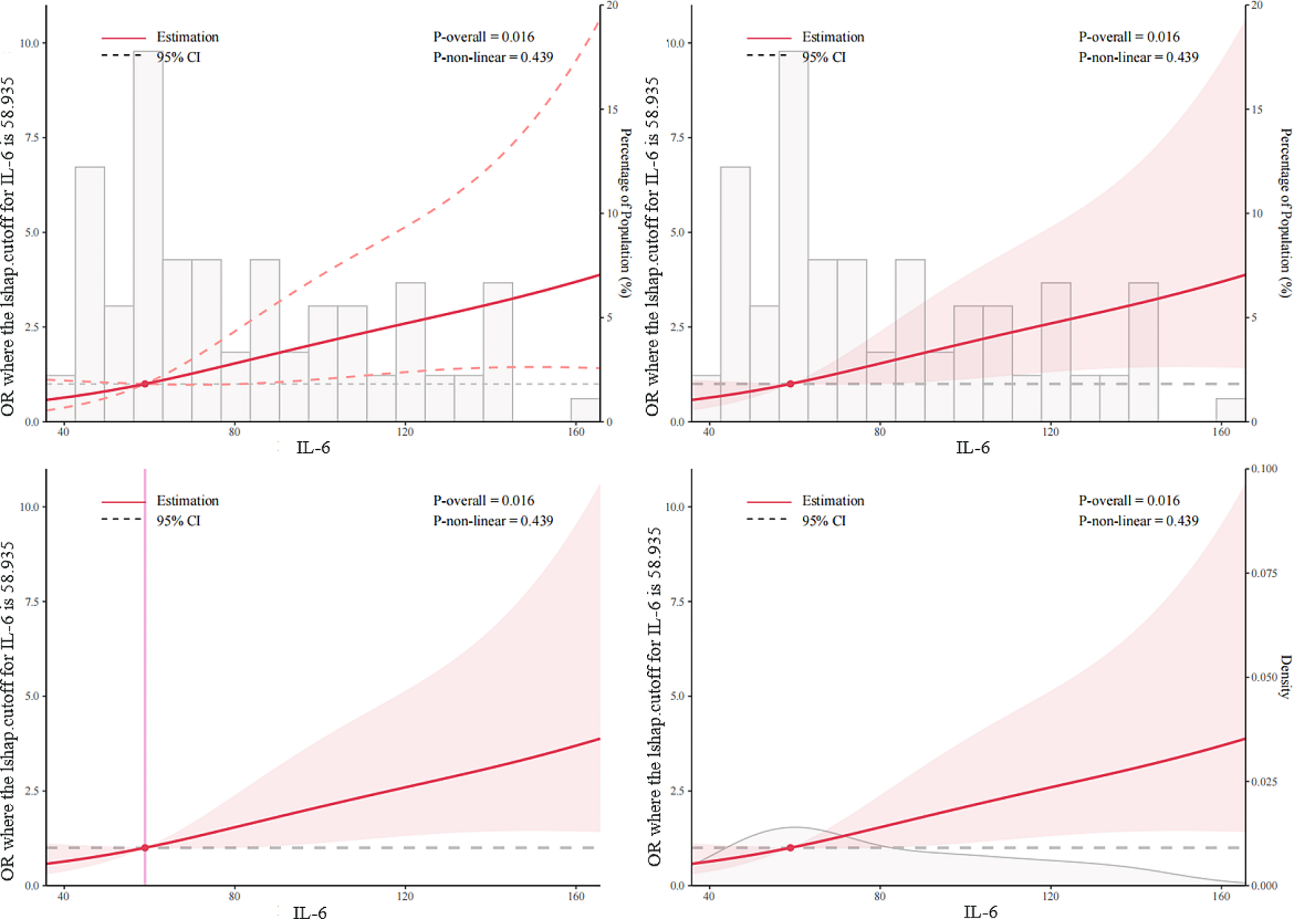
Restricted spline curves for the relationship between IL-6 and POAF in cardiac surgery patients. The red bold line denotes the odds ratio, while the shaded area represents the 95% confidence intervals. *Abbreviations* POAF, postoperative atrial fibrillation; IL-6, interleukin-6; CI, confidence interval

### Combined predictive performance of LAVI and IL-6 for POAF

**Fig. 3:**
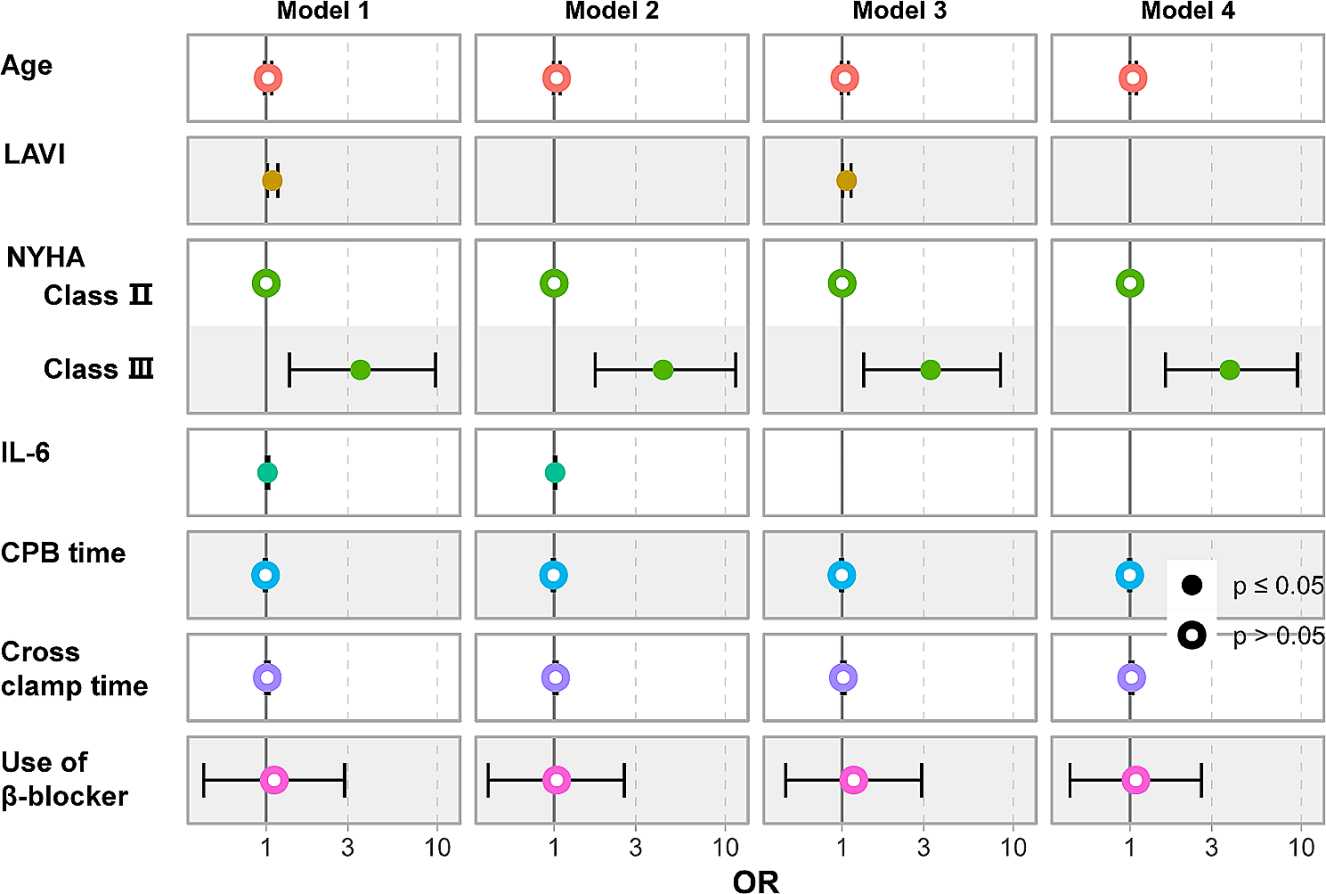
Four predictive models. *Abbreviations* LAVI, left atrial volume index; NYHA, New York Heart Association; IL-6, interleukin-6; CPB, cardiopulmonary bypass; OR, odds ratio

**Fig. 4:**
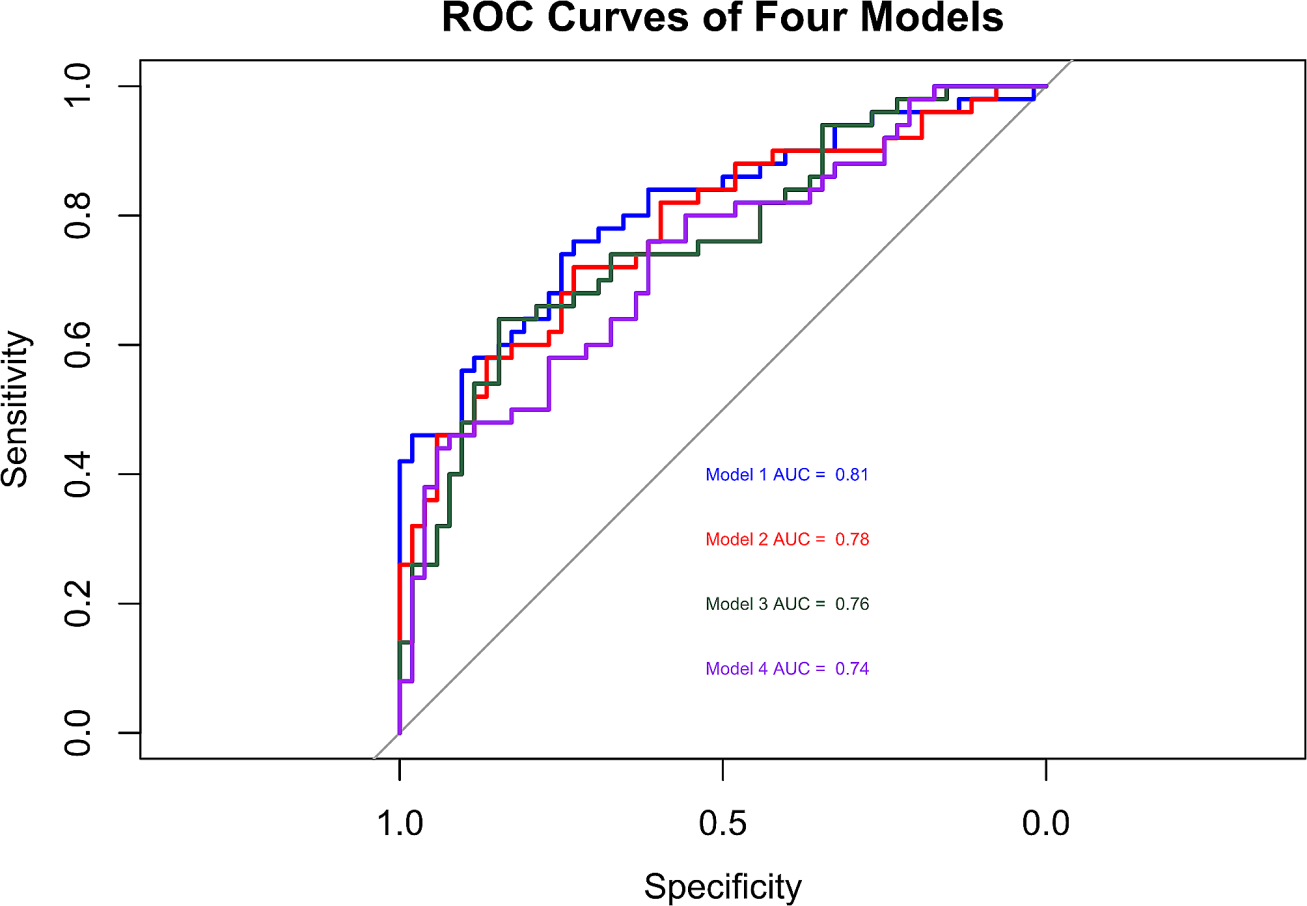
ROC curves of four predictive models. *Abbreviations* ROC, receiver operator characteristic; AUC, area under the curve; LAVI, left atrial volume index; NYHA, New York Heart Association; IL-6, interleukin-6; CPB, cardiopulmonary bypass

In a prospective, randomized study, investigators found that CPB was strongly associated with the incidence of POAF [11]. In a multicenter study, Akintoye E et al. determined that advanced age, prolonged operative time were independent risk factors for POAF [12]. Although debated, β-blocker is the first-line medication in the prevention of POAF [1]. According to these studies and our study, we built 4 predictive models, as shown in Fig. 3. Model 1: age, the preoperative use of β-blocker, LAVI, NYHA, serum IL-6, CPB time and cross-clamp time; Model 2: age, the preoperative use of β-blocker, NYHA, serum IL-6, CPB time and cross-clamp time; Model 3: age, the preoperative use of β-blocker, LAVI, NYHA, CPB time and cross-clamp time; Model 4: age, the preoperative use of β-blocker, NYHA, CPB time and cross-clamp time. The predictive power of each model was quantified by calculating AUCs (Fig. 4). Model 1 had the highest predictive value with an AUC of 0.81. The combined addition of LAVI and IL-6 to Model 1 resulted in an evident increase in the AUC (*p* = 0.03, Table 5). The single addition of LAVI or IL-6 to Model 1 brought a slight increase in the AUCs, but the increase was not statistically significant (both *p* > 0.05). NRIs and IDIs were calculated to evaluate the risk reclassification capability of the models. The incorporation of LAVI and IL-6 did not increase NRIs in all models (all *p* > 0.05), but increased IDIs in all models (all *p* < 0.05), indicating that the combined addition of LAVI and IL-6 may help improve the predictive models’ capacity for risk reclassification.

**Table 5:** Performance metrics of four predictive models

	AUC		Net reclassification improvement	Integrated discrimination improvement
	Index(95%CI)	*p*-value for ΔAUC	Index(95%CI) *p*-value	Index(95%CI) *p*-value
Model 1	0.807(0.723–0.892) Ref.		Ref. Ref.	Ref. Ref.
Model 2	0.779(0.688–0.869) 0.190		-0.002(-0.122-0.119) 0.980	0.056(0.010–0.103) 0.017
Model 3	0.763(0.670–0.855) 0.093		0.019(-0.108-0.145) 0.775	0.074(0.023–0.124) 0.004
Model 4	0.737(0.641–0.834) 0.030		0.096(-0.040-0.232) 0.166	0.1165(0.053–0.180) <0.001

Model 1: age, the preoperative use of β-blocker, LAVI, NYHA, serum IL-6, CPB time and cross-clamp time;

Model 2: age, the preoperative use of β-blocker, NYHA, serum IL-6, CPB time and cross-clamp time;

Model 3: age, the preoperative use of β-blocker, LAVI, NYHA, CPB time and cross-clamp time;

Model 4: age, the preoperative use of β-blocker, NYHA, CPB time and cross-clamp time

*Abbreviations* AUC, area under the receiver operating characteristic curve; CI, confidential interval; LAVI, left atrial volume index; NYHA, New York Heart Association; IL-6, interleukin-6; CPB, cardiopulmonary bypass

## Discussion

Our study confirmed that POAF was a common complication after cardiac surgery with an incidence of up to 49.0%. The occurrence of POAF not only prolonged intubation time, but also contributed to the hemodynamic derangements which increased the need for vasoactive medications. Our results demonstrated that higher levels of preoperative LAVI and postoperative IL-6 were independently associated with an increased risk of POAF in the patients who underwent cardiac surgery. Furthermore, we assessed the combined predictive value of LAVI and IL-6 for POAF, and the results illustrated that the combined use of LAVI and IL-6 improved the predictive performance of models.

Compared with previous studies, our study had both similarities and differences. Previous studies have shown that LAVI was a significant predictor for POAF [4, 13], which was also confirmed by our study. However, Kislitsina et al. found that LAVI had a poor predictive performance with an AUC of 0.66 in respect to evaluate the predictive effect of LAVI for POAF [5]. This study indicated that LAVI alone did not have good predict power. In our study, the optimal cut-off of LAVI for POAF was 29ml/m^2^ and still be considered as normal according to current guidelines [14]. We speculated that this inconsistency may be due to the different measurement methods of LAVI. According to the updated guidelines [14], we used Simpson’s biplane method. And with the improvement of people’s awareness of health, a large number of patients take long-term oral medication to reverse atrial remodeling before surgery.

Gaudino et al. demonstrated that higher IL-6 level was strongly associated with POAF [15], which was also affirmed by our study. In the present study, the optimal cut-off of IL-6 for POAF was 59 pg/ml in the cardiac patients. According to the reference value at our center, the level of IL-6 was significantly elevated after cardiac surgery. This may be because of the influence of cardiopulmonary bypass [16].

The exact mechanism of POAF is not fully understood yet. From the clinical standpoint, it is difficult to make an accurate prediction for POAF. The prevailing view is that the pathogenesis of POAF includes substrates and trigger factors [1]. Upon the basis of the vulnerable substrates of AF, cardiac surgery especially inflammatory response triggers the incident of POAF [17]. Left atrial remodeling is an important underlying substrate for POAF [1, 18]. Enlarged atria reflects the remodeling process, and represents quantifiable surrogate of the arrhythmogenic substrates [19]. The volume of remodeled atria can be reflected by LAVI, which could be measured by echocardiography. Inflammatory response plays another important role in the mechanisms of POAF. In animal experiments, Liu et al. demonstrated that IL-6 elicited early profibrotic properties in the atria via the pSTAT3/STAT3 signaling pathway and contributed to POAF onset [20]. In conclusion, substrates and trigger factors both play essential roles in the development of POAF. We proved that substrates and trigger factors could achieve better predictive performances in our study from the clinical viewpoint.

Our research suggested that combined of LAVI and IL-6 improved the predictive performance of POAF prediction models. These findings may help to identify patients at risk for POAF at an early stage and use potential prophylactic treatments to reduce the incidence of POAF. For those patients with large left atrial before cardiac surgery, drugs reversing left atrial remodeling may prevent the occurrence of POAF. Drugs regulating the inflammatory response may be associated with the decreased incidence of POAF. It is noteworthy that the combined use of the drugs may make the preventive effect better for those patients with enlarged left atrial and excessive inflammatory response simultaneously. These remain to be investigated in future studies. β-blocker is currently the agent choice for pharmacological prophylaxis for POAF in the setting of cardiac surgery [1]. According to recommended guidelines, unless there was a clinical contraindication, β-blocker should be started or continued before cardiac surgery [21]. However, this preventive intervention remains controversial. In our study, the use of β-blocker did not reduce the occurrence of POAF, which may be related to the limited samples. In addition, the timing of reintroduction of β-blocker may also limit the preventive value among patients among patients on long-term oral β-blocker. More treatments focusing on reversing left atrial remodeling and mitigating inflammatory responses should be further investigated.

Our study had several strengths. Innovatively, we proved that the combination of LAVI and IL-6 improved the predictive capabilities for POAF. In the present study, predictors were evaluated both pre- and post-operatively. In addition, we built several predictive models for POAF to illustrate the combined predictive value of ultrasound parameter and blood biomarker for POAF. Additionally, we adjusted several confounding risk factors to determine the association between LAVI and IL-6 and POAF. Nevertheless, some limitations must be acknowledged. Firstly, the sample size in our study was relatively small and our study was based on a single center, which may limit the generalizability of the results of this study. Larger, multicenter studies should be performed to further confirm the results in the next step. If possible, model validation can be performed on other independent datasets. Secondly, it was an observational design precluding causal interpretation of the associations. Finally, the follow-up time was relatively short. We did not investigate the association between predictors and cardiovascular adverse events.

## Conclusions

Our results demonstrated that higher levels of preoperative LAVI and postoperative IL-6 were independently associated with increased risk of POAF in a population of patients undergoing cardiac surgery, indicating that simultaneous use of two markers containing echocardiographic and biological may help to better predict POAF. This study highlighted the importance of an integrated approach, which was useful to better understand the pathogenesis of POAF.

## Data Availability

The datasets generated and/or analyzed during the current study are not publicly available due to the protection for the patient’s privacy but are available from the corresponding author on reasonable request.
